# Conceptions of the pathophysiology of happy hypoxemia in COVID-19

**DOI:** 10.1186/s12931-021-01614-1

**Published:** 2021-01-08

**Authors:** Sebastiaan Dhont, Eric Derom, Eva Van Braeckel, Pieter Depuydt, Bart N. Lambrecht

**Affiliations:** 1 Department of Internal Medicine and Pediatrics, Ghent University, Corneel Heymanslaan 10, 9000 Ghent, Belgium; 2 Department of Respiratory Medicine, Ghent University Hospital, Ghent, Belgium; 3 Department of Intensive Care Medicine, Ghent University Hospital, Ghent, Belgium; 4 VIB-UGent Center for Inflammation Research, Ghent, Belgium

**Keywords:** COVID-19, Happy hypoxemia, Respiratory failure, Gas exchange, Cytokine storm

## Abstract

In their letter-to-the-editor entitled “Misconceptions of pathophysiology of happy hypoxemia and implications for management of COVID-19”, Tobin et al. (Respir Res 21:249, 2020) debated our views on happy hypoxemia in COVID-19 (Respir Res 21:198, 2020). We thank the authors for their interesting comments and alternative viewpoints, and we would like to clarify several important aspects raised.

## Letter

We read with interest the letter-to-the-editor by Tobin, Jubran and Laghi concerning our article on happy hypoxemia in COVID-19 [1, 2]. The authors challenged our narrative review with some interesting thoughts.

We agree with Tobin et al. [1] that “the essential point about happy hypoxemia is that patients can be profoundly hypoxic and yet exhibit no abnormality in breathing pattern”. This is worrying indeed, as the severity of hypoxemia is independently associated with in-hospital mortality and in particular of concern among patients with chronic compromised organ perfusion such as ischemic heart disease [3–5]. However, in our experience, subtle changes in breathing pattern may serve as early warning signs of impending hypoxemic respiratory failure in COVID-19. The respiratory drive shifts minimally in mild hypoxemia, but when arterial oxygen tension drops below a critical threshold, a rise in minute volume does occur [6–9]. That is why we stressed the importance of interpreting pulse oximetry results in the light of the respiratory rate. The oxyhemoglobin-dissociation curve may indeed shift to the right when COVID-19 disease progresses [1]. Although mainly a theoretical concept—some authors claim a leftward shift [10]—the mechanisms and importance of this phenomenon require further investigation.

Tobin and colleagues [1] state that there is no evidence for patient self-inflicted lung injury (P-SILI), neither in COVID-19 nor in other conditions leading to acute respiratory failure. In 2010, Papazian et al*.* revealed that early administration of neuromuscular blocking agents improved 90-day survival in ARDS [11]. The mechanisms underlying this beneficial effect remain controversial, but the potential risk of spontaneous breathing efforts (also investigated in non-intubated patients [12, 13]) and the subsequent need for prevention of these efforts during mechanical ventilation have been highlighted and extensively discussed. Experimental observations have pointed out that P-SILI in humans can lead to pulmonary edema following airway obstruction, due to large variations in pleural pressure [14, 15]. This mechanism may also be responsible for pulmonary edema resulting from re-expansion of a pneumothorax and edema formation in a minority of patients experiencing a severe asthma attack [16, 17]. Additionally, the spontaneous occurrence of pneumomediastinum in non-intubated patients with COVID-19 may be an example of a so-called self-inflicted lung injury [18, 19]. In closing, we believe that P-SILI remains an interesting hypothesis in the disease progression of COVID-19 [20]. Otherwise, we agree with Tobin and colleagues [1, 21] that there is insufficient evidence to warrant premature intubation in order to prevent P-SILI in COVID-19, and that there are much simpler measures to tackle (happy) hypoxemia, such as providing supplemental oxygen.

Tobin et al*.* [1] ask if there is evidence indicating that specific measures will ameliorate cytokine storm and if tackling these targets will benefit COVID-19 patients. Although thoughts on immunomodulatory therapeutic targets in COVID-19 were only mentioned in the margin of our review on happy hypoxemia, data on the role of cytokine release syndrome (CRS) have been increasingly reported since. Increased levels of pro-inflammatory cytokines (e.g., IFNα, IFNγ, IL-1β, IL-6, IL-12, IL-18, IL-33, TNFα, TGFβ) and chemokines (e.g., CXCL10, CXCL8, CXCL9, CCL2, CCL3, CCL5) are associated with pulmonary inflammation and ARDS in patients infected by SARS and MERS-CoV infections [22, 23]. Huang et al*.* reported that COVID-19 patients also show high titers of these cyto- and chemokines and, more importantly, the cytokine release syndrome (CRS) seem to emerge as a main factor driving a more severe clinical course [24]. A multicenter study in China revealed that higher levels of serum IL-6 were correlated with fatal outcome in COVID-19 [25]. Similar studies by Gao et al*.* and Chen et al*.* reported that levels of IL-10, IL-6 and TNF-α were higher in severe COVID-19 [25, 26]. The main contributors to the interplay of the CRS seem to be IL-1, IL-6 and TNF-α and several biological agents targeting these cytokines are being extensively studied in COVID-19 [27]. Tocilizumab, for example, is a humanized monoclonal antibody that binds IL-6 receptors and has proven to be valuable in treatment of cytokine storm triggered by chimeric antigen receptor T-cell therapy [27]. Encouraging results have been reported on its use in severe COVID-19 on length of ICU stay, ventilation-free survival, and also on oxygenation parameters [27, 28]. These findings explain why we eagerly await the final results of ongoing international randomized trials, that not only look at blockade of individual cytokines, but also at combined IL-1 and IL-6 blockade (e.g. NCT04320615, NCT04372186, NCT04331808 and NCT04330638) [28].

In conclusion, we thank the authors for their critical comments. Our main intention was to provide a thought-provoking review on the potential mechanisms leading to the phenomenon of happy hypoxemia in COVID-19, more than providing therapeutic guidelines, which should always be based on scientific evidence.

## Data Availability

Not applicable.

## Abbreviations

COVID-19: Coronavirus disease 2019
MERS: Middle East Respiratory Syndrome
SARS: Severe Acute Respiratory Syndrome Coronavirus
ICU: Intensive Care Unit
P-SILI: Patient self-inflicted lung injury
ARDS: Acute respiratory distress syndrome
IL: Interleukin
TNF: Tumor necrosis factor
TGF: Transforming growth factor
CXCL: C-X-C motif chemokine ligand
CCL: Chemokine (C–C motif) ligand
